# Large Piezoelectric Response and High Carrier Mobilities Enhanced via 6s^2^ Hybridization in Bismuth Chalcohalide Monolayers

**DOI:** 10.3390/nano15241877

**Published:** 2025-12-14

**Authors:** Jing Shi, Chang Han, Haibo Niu, Youzhang Zhu, Yachao Liu, Vei Wang

**Affiliations:** 1 Engineering Research Center of Photovoltaic Technologies and Systems-Universities of Shaanxi Province, Department of Physics, Xi’an Jiaotong University City College, Xi’an 710018, China; yzh_zhu@xjtu.edu.cn; 2 Qingdao Advanced Manufacturing Powder Engineering Research Center, Qingdao R&D Institute, Xi’an Jiaotong University, Qingdao 266330, China; 3 MOE Key Laboratory for Nonequilibrium Synthesis and Modulation of Condensed Matter, School of Physics, Xi’an Jiaotong University, Xi’an 710049, China; 4 Department of Applied Physics, Xi’an University of Technology, Xi’an 710054, China; liuyachao@xaut.edu.cn (Y.L.);

**Keywords:** BiXY monolayers, DFT calculations, piezoelectric property, optical property

## Abstract

In this study, we systematically investigated the piezoelectric and carrier transport properties of two-dimensional (2D) Bi-based chalcohalide monolayers (BiXY, X = Se, Te; Y = Br, I) using first-principles calculations. The phonon dispersion and elastic properties proved that BiXY monolayers are dynamically and mechanically stable. Our results reveal that the stereochemically active 6s^2^ lone-pair electrons of Bi^3+^ play a crucial role in determining the structural and electronic characteristics of these systems. The simultaneous enhancement of Born effective charges and the strong sensitivity of atomic positions to external strain give rise to pronounced piezoelectric responses in BiXY monolayers. Specifically, the calculated piezoelectric coefficients (d_11_) reached 13.16 and 17.76 pm/V for BiSeBr and BiSeI, respectively. The carrier transport properties were estimated using the deformation potential (DP) theory, which yielded upper-bound values under idealized conditions. For instance, in BiTeBr, the effective masses of electrons and holes were 0.15 and 0.40 m_0_, respectively, leading to high carrier mobilities of 2736.1 and 2689.9 cm^2^ V^−1^ s^−1^. These findings highlight the potential of Bi-based chalcohalide monolayers as promising candidates for next-generation multi-functional nanoelectronic and piezoelectric devices.

## 1. Introduction

A substantial number of post-transition metal compounds that contain cations with lone-pair 5s^2^ and 6s^2^ electrons, including Tl^+^, Pb^2+^, Bi^3+^, and Sn^2+,^ have been identified as potential multi-functional materials due to their remarkable properties, such as proper bandgaps, high carrier mobility, strong photocatalytic ability, and extreme tolerance to defects [1,2,3,4]. Such unique characteristics render them highly suitable for diverse technological applications, including chemical sensing platforms, ionizing radiation detectors, and next-generation photovoltaic devices [5,6]. Zhang et al. predicted that the 2D Sn-based Janus SnXY monolayers would be good piezoelectric materials, with the in-plane piezoelectric constant d_11_ of the SnOS monolayer reaching as high as 27.3 pm/V [7]. The halide perovskite CH_3_NH_3_PbI_3_ is widely used as a solar absorber in solar cells, achieving a power conversion efficiency of 20% [8]. In surface-functionalized SnHF monolayers, the electron and hole mobilities are as high as 2520 and 3740 cm^2^ V^−1^ s^−1^, respectively [9]. An ultra-high hole mobility (29,000 cm^2^ V^−1^ s^−1^) was experimentally observed on CVD-grown Bi_2_O_2_Se flakes at a low temperature [10].

The formation of lone-pair ns^2^ electrons depends on the bonding interaction between the s orbitals of the cation and the p orbitals of the anion. The occupied s orbital can be hybridized with the anion p orbital, and the hybridized states present in the upper valence band, leading to the strong dispersion of the valence band. Simultaneously, the conduction band is predominantly composed of spatially extended cation p states, which further hybridize with the anion p states. This cross-bandgap hybridization induces a mixed ionic-covalent bonding character that induces strong lattice polarization and a high static dielectric constant [11,12,13]. In TlCl and TlBr, the Born effective charges are approximately twice the nominal ionic charges and are accompanied by high static dielectric constants (32.70 for TlCl and 30.60 for TlBr) due to cross-gap hybridization between the Tl p states and the Cl/Br p states [14].

Recently, bismuth-based oxyhalides and chalcohalides have been widely studied due to the active lone-pair 6s^2^ electrons and environmentally friendly nature of Bi^3+^ cations. Bismuth-based oxyhalides and chalcohalides have been reported to possess wide potential applications as solar adsorbers, photocatalysis, and radiation detector materials [15,16]. The piezoelectric properties of triple-layered (TL) BiTeX (X = I, Br, Cl) materials are significantly affected by the interlayer van der Waals interaction and present novel negative piezoelectric response (e_33_ = −0.61 C/m^2^ for TL BiTeBr) [17]. Two-dimensional layered Bi_2_SeO_5_ shows strong layer-dependent optical properties, with a piezoelectric stress coefficient e_11_ of 8 × 10^−14^ C/m [18]. BiOX (X = Cl, Br, I) nanomaterials have been demonstrated to exhibit piezocatalytic activity, a synergistic phenomenon arising from the coupling of piezoelectric and catalytic properties. In sheet-like BiOCl nanostructures, mechanical deformation induces piezoelectric polarization, generating abundant free polarized electrons on the surface that participate in redox reactions [19,20]. Theoretical calculations show that bulk BiSeBr and BiSI have appropriate bandgap values in the range of 1.0 to 1.7 eV, as well as smaller effective electron and hole masses [21].

To date, research on bismuth chalcohalides has been largely confined to their three-dimensional bulk forms or catalytic applications, whereas systematic first-principles investigations of piezoelectric coupling and intrinsic carrier transport in their two-dimensional (2D) monolayer forms remain scarce. Given the intrinsic structural asymmetry, strong spin–orbit coupling, and favorable band dispersion inherent to these Janus systems, a comprehensive understanding of their 2D-specific piezoelectric and carrier transport properties is essential to promote their potential in next-generation nanodevices.

In this work, the 6s^2^ hybridization-enhanced piezoelectric and carrier mobility properties of Bi chalcohalide BiXY (X = Se, Te; Y = Br, I) monolayers were studied via DFT calculations. Bi is an ns^2^ lone-pair element with 6s^2^ electrons, which can be hybridized with the p-orbital of X and Y anions in BiXY monolayers. Our study revealed the significant influence of orbital hybridization on the piezoelectric response and carrier mobility properties. The e_11_ was decomposed into electronic contributions and the internal strain term to explore the origin of the large piezoelectric response in the BiXY monolayers, and their contributions were further analyzed. The BiXY monolayers are good piezoelectric materials, with an optimal bandgap range (1.0–1.7 eV) for solar absorption, rendering them as promising candidates for multi-functional energy conversion applications.

## 2. Calculation Methods

All first-principles calculations were carried out within the framework of density functional theory (DFT), as implemented in the Vienna Ab initio Simulation Package (VASP) [22,23]. A plane-wave cut-off energy of 500 eV was employed. A vacuum spacing of 20 Å was introduced along the out-of-plane direction to eliminate interactions between periodic images. The Brillouin zone was sampled using a Γ-centered 16 × 16 × 1 Monkhorst–Pack k-point mesh. The Perdew–Burke–Ernzerhof (PBE) functional within the generalized gradient approximation (GGA) was adopted for the exchange–correlation potential. Given the presence of the heavy Bi atom, spin–orbit coupling (SOC) was explicitly included in all calculations [24]. The total energy convergence threshold for ionic relaxation was set to 10^−5^ eV between successive steps. The band structure and projected density of states (PDOS) were further refined using the Heyd–Scuseria–Ernzerhof (HSE06) hybrid functional with SOC [25]. The VASPKIT code was used for the treatment of the electronic properties [26]. The Berry phase method was used for the polarization calculation [27].

## 3. Results and Discussions

**Figure 1:**
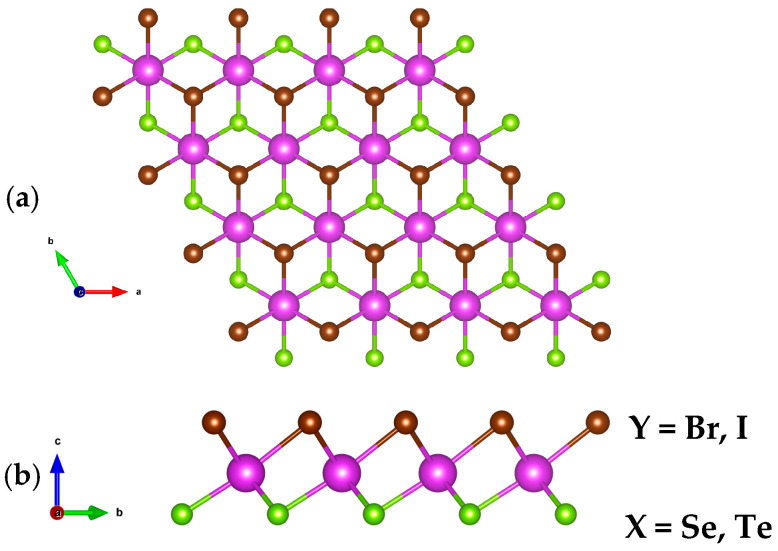
(**a**) Top and (**b**) side views of the BiXY monolayers.

BiXY monolayers (X = Se, Te; Y = Br, I) adopt a Janus configuration, characterized by atomically thin two-dimensional crystals with broken out-of-plane mirror symmetry. Such structures typically follow an MXY stoichiometry, where M denotes a transition metal and X ≠ Y represent distinct chalcogen or halogen atoms. The fully relaxed atomic geometries of the BiXY monolayers (X = Se, Te; Y = Br, I) are depicted in Figure 1. In these Janus-type arrangements, the Bi atomic layer is sandwiched asymmetrically between distinct chalcogen (X) and halogen (Y) planes, giving rise to a non-centrosymmetric structure that belongs to the polar C_3_ᵥ point group. Critically, the absence of both inversion and horizontal mirror symmetries in C_3_ᵥ allows for a non-vanishing spontaneous polarization. Displacement of Bi relative to the X and Y atoms occurred under applied strain, thereby modulating the net polarization and generating non-zero e_11_ and e_31_ piezoelectric coefficients. The optimized in-plane lattice constants were 4.187 Å (BiSeBr), 4.270 Å (BiSeI), 4.345 Å (BiTeBr), and 4.422 Å (BiTeI). For completeness, the corresponding bond lengths and layer thicknesses are provided in Table 1, allowing direct comparison across the different compositions.

**Figure 2:**
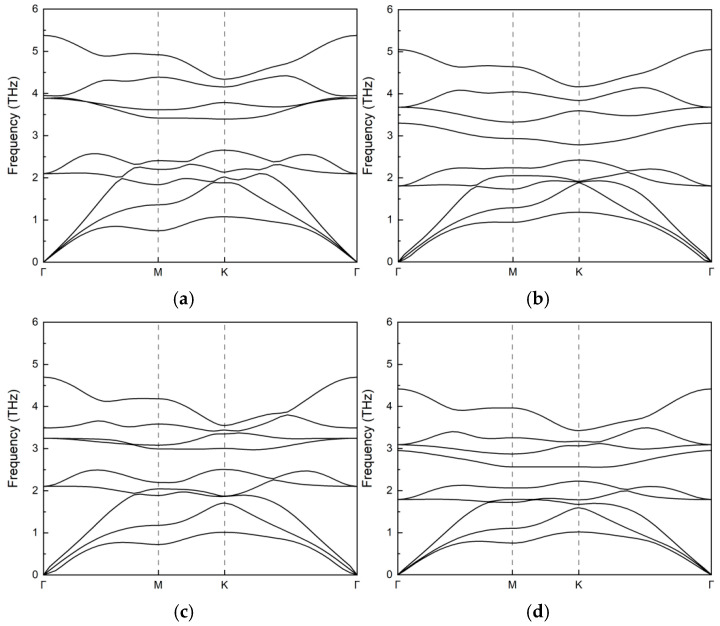
Phonon dispersions of (**a**) BiSeBr, (**b**) BiSeI, (**c**) BiTeBr, and (**d**) BiTeI monolayers.

The dynamic stability of the BiXY monolayers was studied according to the phonon dispersion properties. The calculated phonon dispersions of the BiXY monolayers are presented in Figure 2. The dispersions exhibited fully stable vibrational modes across the Brillouin zone, as evidenced by the absence of imaginary modes, confirming their intrinsic dynamic stabilization.

**Figure 3:**
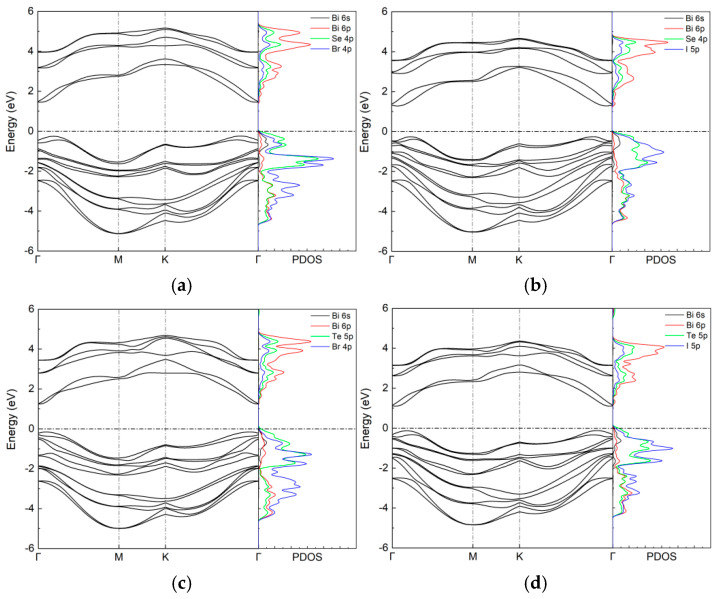
Band structures and PDOS of (**a**) BiSeBr, (**b**) BiSeI, (**c**) BiTeBr, and (**d**) BiTeI monolayers calculated using HSE06 + SOC.

The electronic properties of the BiXY monolayers were studied using HSE06 + SOC calculations. The bandgaps of the BiSeBr, BiSeI, BiTeBr, and BiTeI monolayers were 0.96, 0.87, 0.83, and 0.65 eV, respectively, calculated using the PBE + SOC method. Since the PBE + SOC method usually underestimates the bandgap values, we further calculated the band structures with the HSE06 + SOC method. In previous studies, the bandgaps of bulk bismuth oxyhalides and chalcohalides computed using the HSE06 + SOC method were in excellent agreement with experimental values [21,24]. The band structures of the BiXY monolayers calculated using the HSE06 + SOC method are presented in Figure 3. The bandgaps of the BiSeBr, BiSeI, BiTeBr, and BiTeI monolayers increased to 1.62, 1.45, 1.39, and 1.18 eV, respectively. The predicted bandgaps of the BiSeBr and BiSeI monolayers were in good agreement with the experimentally measured values for their bulk form (1.54 eV for bulk BiSeBr and 1.29 eV for bulk BiSeI) [28,29,30]. The bandgaps of the BiXY monolayers were in the optimal range (1.0–1.7 eV) as potential solar absorber materials [21,31,32].

Based on the projected density of states (PDOS) analysis, the conduction band minimum (CBM) was predominantly derived from the p orbitals of Bi atoms, whereas the valence band maximum (VBM) was mainly governed by the p orbitals of the X and Y atoms. In addition, the Bi 6s and 6p orbitals exhibited a non-negligible contribution to the VBM, reflecting the presence of additional orbital hybridization. The interaction between the fully occupied cation s states and the anion p states near the VBM produced a dispersive valence band which, in turn, reduced the effective mass of holes. Furthermore, the spatially extended Bi p orbitals strongly hybridized with the anion p states, leading to pronounced cross-bandgap hybridization. This interaction imparted a mixed ionic–covalent bonding character, which was closely associated with enhanced lattice polarization, anomalously large Born effective charges, and an increased static dielectric constant. Such electronic features are widely recognized as favorable characteristics in ferroelectric materials [33,34].

The calculated Born effective charges $Z_{11}^{*}$ of the BiXY monolayers are summarized in Table 2. The results reveal that the Born effective charges of Bi, X, and Y atoms were significantly enhanced compared with their nominal valence charges, indicating strong dynamical charge transfer and pronounced lattice polarization effects. The Born effective charges of Bi and Br were +5.56 e and −2.83 e in the BiSeI and BiTeBr monolayers, respectively, which were significantly larger than their nominal chemical valences of +3 e and −1 e, respectively. The Born effective charges play an important role in ferroelectric and piezoelectric properties; large Born effective charges usually mean strong orbital hybridization, accompanied by large piezoelectric constants [14,35,36].

The elastic properties of the BiXY monolayers were examined via the evaluation of the independent elastic constants C_11_, C_22_, and C_12_, together with the derived mechanical parameters, such as Young’s modulus (Y_2D_) and Poisson’s ratio (*ν*). The numerical values are presented in Table 3. The calculated elastic constants covered the Born–Huang stability criteria (C_11_ > 0, C_11_ > |C_12_|), indicating that the BiXY monolayers were mechanically stable [37]. In Table 3, the BiXY monolayers show smaller elastic constants. The C_11_ values of BiSeBr, BiSeI, BiTeBr, and BiTeI were 31.37, 30.40, 28.10, and 26.85 N/m, respectively. Young’s moduli of BiSeBr, BiSeI, BiTeBr, and BiTeI were 28.8, 27.5, 26.3 and 24.9 N/m, respectively, indicating good flexibility of the BiXY monolayers. Young’s moduli of the BiXY monolayers were significantly smaller than those of graphene (343.0 N/m), MoS_2_ monolayer (120.0 N/m), and BP monolayer (135.7 N/m), and comparable to that of black phosphorus (21.9 N/m) [38,39,40]. The small Young’s modulus of the BiXY monolayer indicates its potential applications for high-performance mechanically flexible nanoelectronics [41,42,43].

**Figure 4:**
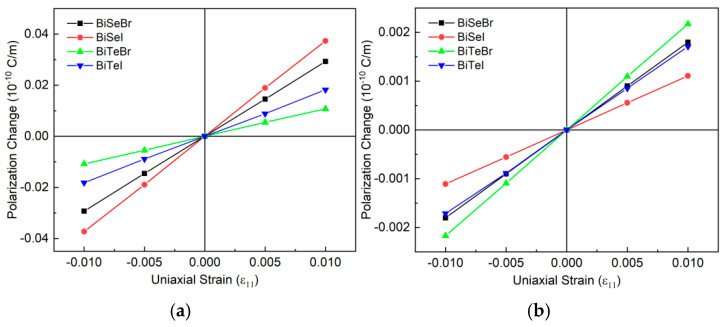
(**a**) In-plane and (**b**) out-of-plane polarization change vs. the uniaxial strain applied in the x-direction. The piezoelectric coefficients e_11_ and e_31_ were obtained using linear fitting method.

The piezoelectric constants of the Janus BiXY monolayers were calculated using the linear fitting method, as shown in Figure 4. The calculated e_11_ and e_31_, as well as the corresponding d_11_ and d_31_, are listed in Table 4. The BiSeI monolayer showed the largest in-plane e_11_ of 3.73 × 10^−10^ C/m, and the corresponding d_11_ was as large as 17.76 pm/V. The BiTeBr monolayer showed the smallest e_11_ of 1.08 × 10^−10^ C/m, and the corresponding d_11_ was 5.15 pm/V. The BiXY monolayers showed larger d_11_ values due to their relatively smaller elastic constants, C_11_ and C_12_. As summarized in Table 4, the calculated in-plane piezoelectric coefficient (d_11_) of the BiSeI monolayer was considerably larger than that of many reported piezoelectric materials, such as bulk α-quartz (2.31 pm/V), wurtzite-AlN (5.44 pm/V), wurtzite-ZnO (11.67 pm/V), and two-dimensional MoS_2_ (3.65 pm/V) [44,45,46,47]. This pronounced in-plane piezoelectricity highlights BiSeI as a promising candidate among emerging two-dimensional piezoelectric materials.

The in-plane piezoelectric constant e_11_ in BiXY monolayers was decomposed into two components, the electronic and ionic contributions, to further clarify the underlying mechanisms responsible for the origin of the large piezoelectric constants e_11_ in the BiXY monolayers [48,49]:

$$e_{11}=e_{11}^{ele}+\sum_{i}\frac{ea}{A}Z_{11}^{*}\left(i\right)\frac{du_{1}(i)}{d\eta_{1}}$$

In this expression, the first term $e_{11}^{ele}$ represents the purely electronic contribution to the total piezoelectric coefficient, obtained from clamped-ion calculations where all atoms are fixed at their equilibrium positions under zero strain. The second term corresponds to the ionic contribution, which arises from the internal relaxation of atoms in response to strain. Specifically, the displacement of the i-th atom in the x-direction, denoted as ($u_{1}(i)$), under an applied macroscopic strain ($\eta_{1}$), is quantified by the derivative $\frac{{du}_{1}(i)}{d\eta_{1}}$.

As shown in Table 5, the BiSeBr and BiSeI monolayers presented a larger electronic contribution $e_{11}^{ele}$ than the BiTeBr and BiTeI monolayers. The BiSeI monolayer showed the largest $e_{11}^{ele}$ of 1.91 × 10^−10^ C/m, which was almost five times that of the BiTeI monolayer (0.40 × 10^−10^ C/m). The BiTeBr monolayer showed a negative electronic contribution $e_{11}^{ele}$ of −0.42 × 10^−10^ C/m, resulting in the smallest total piezoelectric constant e_11_. The BiSeBr and BiSeI monolayers showed a larger ionic contribution $e_{11}^{ion}$ to the total piezoelectric constant e_11_, which can be calculated using the Born effective charges $Z_{11}^{*}(i)$ and $\frac{{du}_{1}(i)}{d\eta_{1}}$, than the BiTeBr and BiTeI monolayers. The BiSeBr monolayer presented the largest $e_{11}^{ion}$ of 1.89 × 10^−10^ C/m. The smaller $e_{11}^{ion}$ of the BiTeBr and BiTeI monolayers was attributed to the smaller $\frac{{du}_{1}(Bi)}{d\eta_{1}}$. The $\frac{{du}_{1}(Bi)}{d\eta_{1}}$ in the BiSeI monolayer was 0.047, which was almost three times that in the BiTeBr monolayer (0.016). The enhanced piezoelectric response in the BiSeBr and BiSeI monolayers can be attributed to the substantial Born effective charge of Bi atoms combined with their pronounced ionic displacement capability, which resulted in an enhanced sensitivity of Bi atoms to the internal strain.

The band structure calculations show that the BiXY monolayers possess suitable optical bandgaps for solar absorber applications (1.0–1.7 eV). In solar absorber materials, a reduced effective mass of charge carriers is highly desirable, as it is essential for achieving high carrier mobility. The effective mass of electrons ($m_{e}^{*}$) and holes ($m_{h}^{*}$) in the Γ–X direction was calculated based on HSE06 + SOC, and the results are listed in Table 6. The studied BiXY monolayers possessed low hole and electron masses due to the strong 6s^2^ hybridization effect. The electron effective masses of BiSeBr and BiSeI were 0.20 and 0.23 m_0_, respectively, comparable to those of MoSI (0.204 m_0_) and halide perovskite CH_3_NH_3_PbI_3_ (0.23 m_0_) [50,51,52]. Meanwhile, the electron effective masses of BiTeBr and BiTeI were even lower, at 0.15 and 0.16 m_0_, respectively, comparable to that of bulk Si (0.19 m_0_) [53].

High carrier mobility is crucial for high-performance solar absorber devices. The carrier mobility properties of the BiXY monolayers were estimated using the deformation potential (DP) theory [54,55]:

$$\mu =\frac{e{ћ}^{3}C_{11}}{K_{B}T{m^{*}m_{d}^{*}E}_{1}^{2}}$$

where $m^{*}$ represents the effective masses in the x-direction, $m_{d}^{*}$ represents the average effective masses in the x- and y-directions, and $E_{1}$ is the deformation potential constant. DP theory considers only intrinsic carrier scattering by acoustic phonons and, therefore, provides an upper-bound estimate of carrier mobility. The calculated results are listed in Table 6. The BiTeBr monolayer presented the largest electron and hole mobilities of 2736.1 and 2689.9 cm^2^ V^−1^ s^−1^, respectively. The electron mobility of the BiTeBr monolayer was larger than those of MoS_2_ (130 cm^2^ V^−1^ s^−1^), phosphorene (1000 cm^2^ V^–1^ s^–1^), bulk silicon (1350 cm^2^ V^−1^ s^−1^), and MAPbI_3_ (1500 cm^2^ V^−1^ s^−1^) [56,57,58]. The relaxation time $\tau =\mu m^{*}/e$ of the carriers was calculated. The carrier relaxation time is a critical parameter in evaluating the performance of light-absorbing materials for optoelectronic applications, particularly in photovoltaic devices. In solar absorber materials, longer carrier relaxation times facilitate greater diffusion lengths, which are vital for efficient charge transport across the active layer.

**Figure 5:**
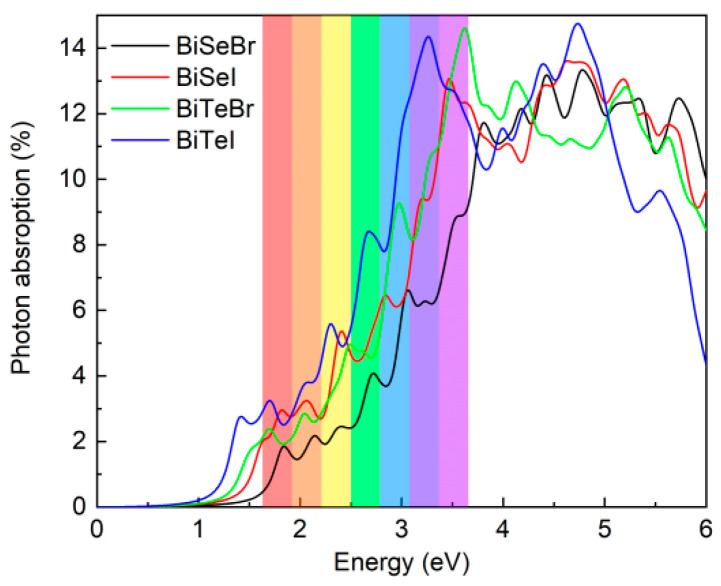
Calculated optical absorption spectra of the BiXY monolayers.

BiXY monolayers possess suitable bandgaps and low carrier effective masses, rendering them promising candidates for light-harvesting applications, such as photovoltaics and photocatalysis. We calculated their absorption spectra using the HSE06 hybrid functional to assess their optical response. As shown in Figure 5, the spectra exhibited pronounced peaks within the visible region and significant absorption at higher-energy UV wavelengths. This spectral profile confirmed the capability of BiXY monolayers to efficiently absorb both visible and high-energy UV photons, thereby highlighting their potential for effective solar energy conversion.

## 4. Conclusions

In conclusion, the electronic structures, piezoelectric responses, optical characteristics, and carrier mobilities of Janus BiXY monolayers (X = Se, Te; Y = Br, I) were systematically investigated using first-principles calculations. Phonon dispersion and elastic analyses confirmed their dynamic and mechanical stability, supporting their potential realizability. HSE06 + SOC calculations predicted indirect bandgaps ranging from 1.18 to 1.62 eV, which fall within the optimal window of 1.0–1.7 eV for visible-light optoelectronic applications. Deformation potential theory further suggested potentially high electron mobilities (with an electron mobility up to 2736.1 cm^2^ V^−1^ s^−1^ for BiTeBr), although this estimate represents an upper-bound under idealized intrinsic conditions. While these findings identified BiXY monolayers as a promising Janus material with coexisting piezoelectricity and favorable carrier mobilities, highlighting their potential for applications in multi-functional nanoscale applications such as piezoelectric sensors and energy-harvesting devices, we emphasize that experimental validation (particularly of synthesis feasibility, ambient stability, and carrier transport) is essential before device consideration.

## Figures and Tables

**Table 1:** Structural parameters of the BiXY monolayers, including the lattice constant, thickness, bond lengths of Bi-X/Bi-Y, and the bandgap values.

	a (Å)	h (Å)	Bi-X (Å)	Bi-Y (Å)	PBE + SOC (eV)	HSE06 + SOC (eV)
BiSeBr	4.187	3.464	2.872	3.082	0.96	1.62
BiSeI	4.270	3.643	2.883	3.269	0.87	1.45
BiTeBr	4.345	3.570	3.059	3.098	0.83	1.39
BiTeI	4.422	3.776	3.071	3.286	0.65	1.18

**Table 2:** Born effective charges $Z_{11}^{*}$ and static dielectric constants ԑ_0_ of the BiXY monolayers.

	$Z_{11}^{*}\left(Bi\right)$ (e)	$Z_{11}^{*}\left(X\right)$ (e)	$Z_{11}^{*}\left(Y\right)$ (e)	ԑ_0_
BiSeBr	5.31	−2.90	−2.41	9.92
BiSeI	5.56	−3.34	−2.21	10.70
BiTeBr	5.05	−2.22	−2.83	9.88
BiTeI	5.30	−2.65	−2.64	10.24

**Table 3:** The calculated elastic constants, Young’s moduli, and Poisson’s ratios of the BiXY monolayers.

	C_11_ (N/m)	C_12_ (N/m)	Y_2D_ (N/m)	ν
BiSeBr	31.37	9.03	28.8	0.29
BiSeI	30.40	9.40	27.5	0.31
BiTeBr	28.10	7.12	26.3	0.25
BiTeI	26.85	7.27	24.9	0.27

**Table 4:** Piezoelectric constants e_ij_ and d_ij_ of the BiXY monolayers.

	e_11_ (10^−10^ C/m)	e_31_ (10^−10^ C/m)	d_11_ (pm/V)	d_31_ (pm/V)
BiSeBr	2.94	0.18	13.16	0.45
BiSeI	3.73	0.11	17.76	0.28
BiTeBr	1.08	0.22	5.15	0.63
BiTeI	1.81	0.17	9.24	0.50

**Table 5:** Electronic ($e_{11}^{ele}$) and ionic ($e_{11}^{ion}$) parts of the total piezoelectric constant e_11_.

	$e_{11}^{ele}$ (10^−10^ C/m)	$e_{11}^{ion}$ (10^−10^ C/m)	$\frac{{du}_{1}(Bi)}{d\eta_{1}}$	$\frac{{du}_{1}(X)}{d\eta }$	$\frac{{du}_{1}(Y)}{{d\eta }_{1}}$
BiSeBr	1.04	1.89	0.039	0.111	−0.150
BiSeI	1.91	1.82	0.047	0.108	−0.155
BiTeBr	−0.42	1.50	0.016	0.117	−0.135
BiTeI	0.40	1.41	0.025	0.120	−0.144

**Table 6:** The calculated effective masses of electrons and holes, elastic constant C_11_, deformation potential constant E_1_, charge carrier mobilities μ, and relaxation time τ of the BiXY monolayers at 300 K.

		Effective Mass (m_0_)	C_11_ (N/m)	E_1_ (eV)	Mobility (cm^2^ V^−1^ s^−1^)	Relaxation Time (fs)
BiSeBr	Electron	0.20	31.37	3.53	1342.2	152.8
	Hole	0.45	31.37	1.35	1812.7	464.4
BiSeI	Electron	0.23	30.40	3.86	822.5	107.7
	Hole	0.35	30.40	2.41	911.2	181.6
BiTeBr	Electron	0.15	28.10	3.12	2736.1	233.7
	Hole	0.40	28.10	1.18	2689.9	612.6
BiTeI	Electron	0.16	26.85	3.42	1912.3	174.2
	Hole	0.37	26.85	2.01	1035.3	218.1

## Data Availability

Dataset available on request from the authors.
